# Small angle X-ray scattering study of microvoid evolution and pertinence of microvoid and mechanical properties in γ-irradiated CFs

**DOI:** 10.1039/c7ra11968b

**Published:** 2018-01-09

**Authors:** Tingting Feng, Yufen Zhao, Jie Shi, Liangsen Liu, Nan Li, Zhiwei Xu, Lihuan Zhao, Xu Tian, Wei Mai, Yinglin Li

**Affiliations:** State Key Laboratory of Separation Membranes and Membrane Processes, School of Textiles, Tianjin Polytechnic University Tianjin 300387 China xuzhiwei@tjpu.edu.cn +86 22 83955231 +86 22 83955231

## Abstract

To explore the mechanism of microvoid evolution and the pertinence of microvoid and mechanical behavior of carbon fibers (CFs) in γ-irradiation, T700 CFs were exposed to γ-rays under epoxy chloropropane (ECP) and argon (Ar) at room temperature. The results from small angle X-ray scattering (SAXS) showed that the average microvoid radius of the CFs decreased gradually from 4.8406 nm for pristine fibers to 3.6868 nm (ECP) and 3.4223 nm (Ar), indicating that γ-irradiation could obviously decrease the microvoid in CFs owing to annealing and rearrangement effects. More significantly, active media would enlarge the surface microvoid of fibers, thus the microvoid of CFs irradiated in ECP was overall larger than that in Ar. The tensile strength of CFs was increased from 5.74 GPa for the pristine fibers to 6.78 GPa (Ar) and 6.18 GPa (ECP) for the irradiated CFs along with a decrease in the microvoid. Therefore, this would provide a key to investigate the evolution of the CF microvoid during γ-irradiation, which was conducive to improving the mechanical properties of γ-irradiated CFs.

## Abstract 2

To explore the mechanism of microvoid evolution and the pertinence of microvoid and mechanical behavior of carbon fibers (CFs) in γ-irradiation, T700 CFs were exposed to γ-rays under epoxy chloropropane (ECP) and argon (Ar) at room temperature.

## Graphical abstract

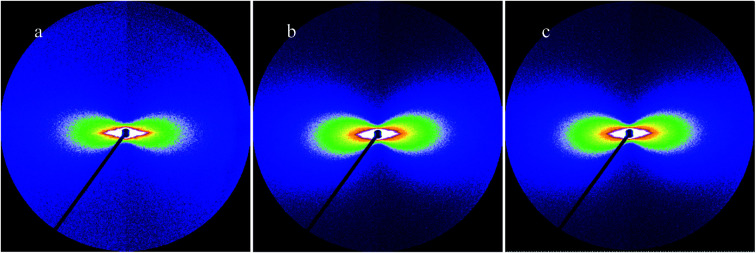

## 1. Introduction

Carbon fibers (CFs) have been widely applied in a variety of applications such as aviation, building and transport and so on owing to their superior mechanical properties.^1^ However, the practical mechanical properties of CFs were far lower than their theoretical value.^2^ Therefore, all kinds of modification treatments were executed such as plasma treatment,^3^ electrostatic spray painting,^4^ electrochemical processing,^5^ γ-irradiation and so on. In recent years, γ-irradiation had attracted tremendous attention because this technology could simultaneously improve surface activity and mechanical performance of CFs. In our previous work,^6^ it had been proved that the surface energy of CFs was improved after irradiation in acrylic acid. Li *et al.*^7^ found that the oxygen/carbon ratio of CFs increased rapidly after irradiation, and improved the surface roughness of CFs by γ-irradiation in air. Xiao *et al.*^8^ thought that the tensile strength and Young's modulus had been improved respectively 17.4% and 16.1% within 300 kGy. Li *et al.*^7^ thought that Young's modulus of CFs was increased 35% within 2 MGy.

Most researchers agreed that improvement of surface activities was deduced by the change of surface functional groups and morphology, but the reason for enhancement of mechanical property is still ambiguous. As is well known, the change of CFs microstructure after γ-irradiation was the fundamental cause of improvement for mechanical property. Xiao *et al.*^8^ deemed that the crosslinking between graphite layers caused by γ-irradiation was the main reason for improvement of mechanical property. Li^9^ and Xu^10^*et al.* reported that the improvement of graphitization degree and optimizing of crystallite size collectively led to the enhance of mechanical property. Obviously, there was not any research in previous literatures to report the evolution of microvoid for γ-irradiated CFs. However, it is well known that the microvoid is also significant for the mechanical property of CFs.^11^ Andreas F. *et al.*^12^ put forward a point that the formation of microvoid was correlated with the cyclization and aromatization reactions of the fibers, which were important steps in the formation of carbon fiber structure. Zhu *et al.*^13^ thought the growth and formation of small microvoid could improve the mechanical performance of CFs. Shioya *et al.*^14^ found that formation of microvoids, which depended on the strength of the linking regions, contributed to the fracture toughness of the fiber. Therefore, the microvoid evolution rules of CFs under γ-irradiation need to be further elucidated.

In this paper, T700CFs were exposed to γ-rays under in epoxy chloropropane (ECP) and argon (Ar) at room temperature. The evolution of microvoid structure for γ-irradiated CFs was characterized by small angle X-ray scattering (SAXS). And the pertinence of microvoid and mechanical property for γ-irradiated CFs had also been evaluated.

## 2. Experimental

### 2.1. γ-irradiation experiments

A bundle of PAN-based CFs (T700) with 1000 filaments purchased from Toray (Japan) was used to investigate the microvoid. The CFs were placed in glass vessel and irradiated in Ar and ECP at room temperature respectively at Tianjin Institute of Technical Physics with ^60^Co γ-rays. The absorbed dose was accumulated to 100 kGy with dose rate of 0.3 kGy h^−1^.

### 2.2. Characterizations

SAXS was measured at beamline BL16B with an X-ray wavelength of 0.124 nm at Shanghai Synchrotron Radiation Facility, China. The CCD (Charge-Coupled Device, Mar165, Mar research, Germany) was equipped at a distance 2000 mm away from the samples. The morphology of fibers before and after irradiation was studied by HitachiS-4800 field-emission (SEM). X-ray diffraction (XRD) was used to characterize crystal structure of CFs. The interval resolution of 2*θ* was 0.02, and X-ray wavelength was 0.15418 nm. Finally, the mechanical tests of fibers were performed in a universal testing machine with a load cell of 10 N, as reported elsewhere.^15^ The specimen was set up to test, by cutting the paper case and using a crosshead speed of 5 mm min^−1^ to break. Not less than 20 filaments for each specimen were tested. Young's modulus was calculated according to stress–strain curve.

## 3. Results and discussion

### 3.1. Microvoid evolution of CFs under γ-irradiation

**Fig. 1:**
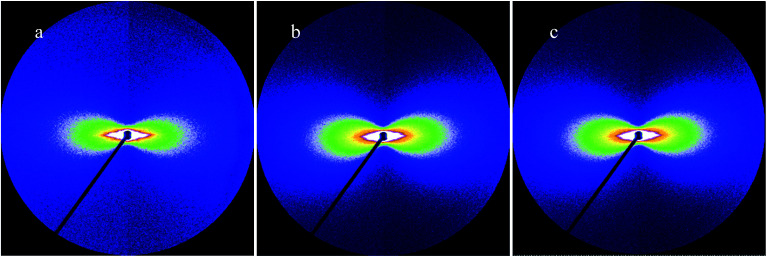
Scattering patterns of CFs at different irradiation media from SAXS (a) As-received, (b) T700-ECP, (c) T700-Ar.

**Fig. 2:**
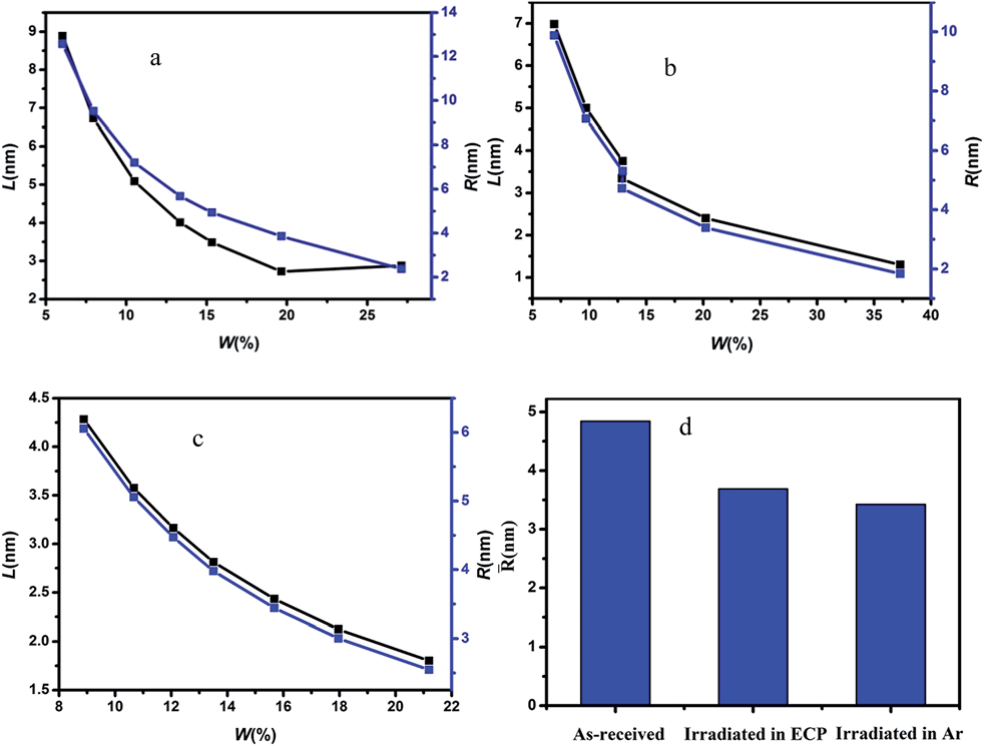
Parameters of microvoid for pristine and irradiated CFs determined by SAXS (a–c) the microvoid length *L* and microvoid radius *R* for pristine and irradiated CFs ((a) as-received, (b) T700-ECP, (c) T700-Ar), (d) the average microvoid radius *R̄* for pristine and irradiated CFs.

**Fig. 3:**
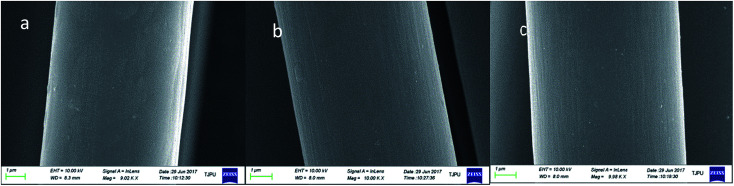
SEM micrographs of pristine and irradiated fiber surface (a) as-received, (b) T700-ECP, (c) T700-Ar.

The scattering patterns of CFs at different irradiation media were exhibited in Fig. 1. The fibers were fixed in vertical direction, and scattering patterns appeared a maximum scattering intensity in horizontal direction, which indicated preferential orientation of the long axes of microvoid in the fibers with respect to the horizontal direction. The microvoid was regarded as the cylinder based on previous literature.^11^ A series of parameters of microvoid were obtained according to Guinier law by Fankuchen gradual tangent method.^16^ The microvoid length *L*, microvoid radius *R*, the proportion of different particle *W* and average microvoid radius *R̄* at different media were shown in Fig. 2. The surface morphology of CFs irradiated in ECP and Ar was observed by SEM, and the results were shown in Fig. 3.

Remarkable differences in morphology could be observed between pristine fibers and irradiated CFs. The outer surface of pristine fibers was smooth, and there almost no obvious groove or protuberance. However, there was slight groove on the outer surface for irradiated CFs owing to the etching effect by γ-rays. What's more, the fibers irradiated in ECP had more groove than that in Ar under the effect of reactive media. *L* of pristine fibers was around 3–9 nm, but minished to 1–7 nm (ECP) and 1–5 nm (Ar) for γ-irradiation CFs. Furthermore, microvoid with radius at 2–6 nm occupied respectively 75.48% (pristine), 83.36% (ECP) and 100% (Ar). *R̄* of CFs irradiated in ECP and Ar respectively was 3.6868 nm and 3.4223 nm, decreasing 23.83% and 25.16% compared with the 4.8406 nm for pristine fibers. These parameters proved that the microvoid of CFs was decreased by γ-irradiation. It could be understood that Compton scattering effect was the main reason for the interaction of γ-rays with CFs. Gamma photons were converted to fast electrons which interacted with microvoid, changing their state with great annealing activation energy.^17^ The drastic swelling occurred in microvoid under the annealing activation energy. When fibers obtained enough annealing energy, the microvoid would collapse owing to the existence of amorphous carbons. Then spare gas in microvoid would be excluded by high energy through the crack in fiber surface caused by etching effect of γ-rays. Consequently, carbon atomic displacement and rearrangement could occur in collapsed microvoid. As a result, the collapsed void transforms into smaller microvoid and volume of microvoid would decrease under combined action of annealing and rearrangement.

More significantly *R̄* of CFs irradiated in Ar was 0.2645 nm smaller than that in ECP, proving that activity media would enlarge the microvoid of CFs surface. Etching effect and grafting effect caused by γ-rays might be mostly responsible for this phenomenon. The γ-irradiation increased the surface activity and disordered the structure of fiber surface due to the increase of oxygen-containing functional groups and grooves of fiber surface by the combined effect of γ-rays and reactive media,^17^ enlarging microvoid size of outer-surface part. However, the media had no impact on the microvoid of sub-surface and core for fibers because the action depth of irradiation media was limited. Hence, microvoid size of CFs irradiated in ECP was larger than that in Ar.

### 3.2. Crystallite structure evolution of CFs under γ-irradiation

**Fig. 4:**
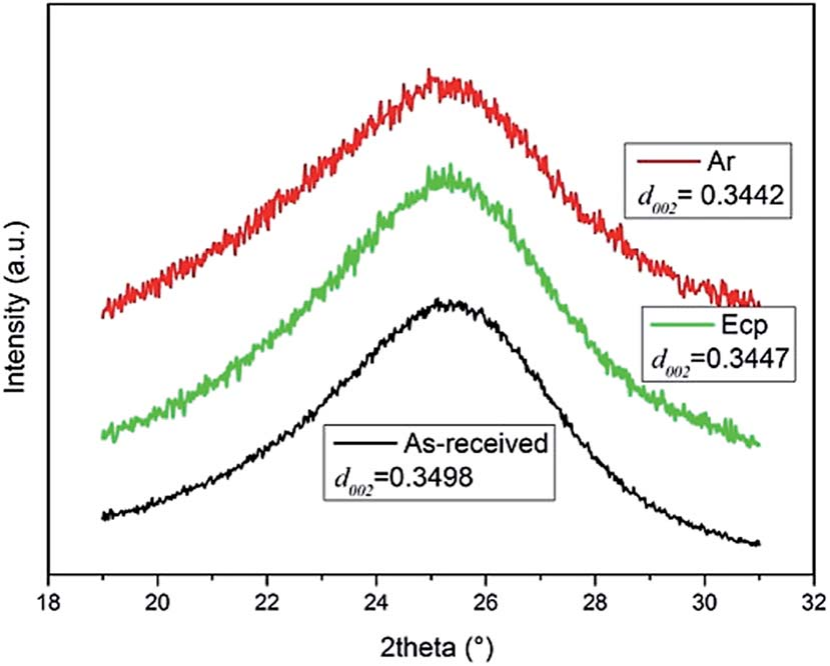
X-ray diffraction profiles of pristine and irradiated CFs (the inset show the magnified 002 peak in the range of 21–30°).

The XRD patterns of irradiated CFs were demonstrated in Fig. 4. The main peak of all samples occurred at about 2*θ* ≈ 25.5°, corresponding to the (002) lattice plane. The Bragg equation was used to calculate interlayer spacing to explore the transformation of crystal structure for CFs.^18^ The average *d*_002_ interlayer spacing decreased gradually from 0.3498 nm for the pristine fibers to 0.3442 nm (Ar) and 0.3447 nm (ECP) for the irradiated CFs, which was closer to the 0.3354 nm for hexagonal graphite, indicating the improvement of the graphitization degree of irradiated CFs.^19^ The decrease of *d*_002_ interlayer spacing revealed that degree of graphitization was improved due to the Compton scattering effect caused by γ-rays.^20^ The *d*_002_ of CFs irradiated in ECP was larger than that in Ar due to the surface grafting reactions. Oxygen-containing functional groups were significantly introduced into the graphene planes in outer-surface part of CFs irradiated in ECP by the combined effect of reactive media and γ-rays, which leaded to the decrease of graphitization degree for outer surface part.^21^ But *d*_002_ of sub-surface and core for fibers was not influenced by media. Therefore, the graphitization degree of CFs irradiated in ECP was lower than that in Ar.

### 3.3. Pertinence of mechanical property and microvoid evolution for γ-irradiated CFs

**Fig. 5:**
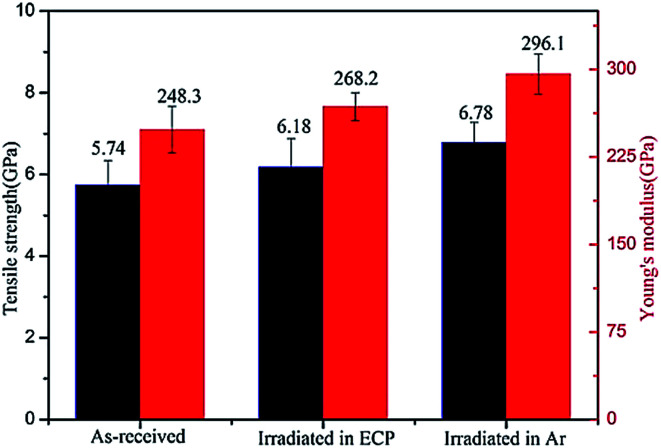
Tensile strength and Young's modulus of pristine and irradiated CFs in different media.

Fig. 5 showed the tensile strength of CFs increased from 5.74 GPa for the original fibers to 6.78 GPa (Ar) and 6.18 GPa (ECP) for the irradiated CFs, resulting in 18.1% and 7.6% improvement, respectively. Young's modulus for CFs were 248.3 GPa (original fibers), 299.1 GPa (Ar) and 268.2 GPa (ECP), increasing 20.4% (Ar), and 8.0% (ECP) after irradiation. Mechanical property of the fibers irradiated in Ar had remarkable advantage over that in ECP. Li^9^ and Xu^10^*et al.* had reported that improvement of mechanical property was caused by the microstructure evolution under γ-irradiation such as the improvement of graphitization degree and the decrease of *d*_002_ interlayer spacing. In our previous work,^22^ the viewpoint that different active media had slight influence on the CF *I*_D_/*I*_G_, indicative of the degree of graphitization, had been proved. In this work, there was no obvious change for *d*_002_ in different active media. Oppositely, the active medium (ECP) had remarkable influence to microvoid. Meanwhile, evolution of mechanical property in different irradiation media was consistent with the evolution of microvoid. Therefore, we could conclude that mechanical property of CFs was enhanced along with the decrease of microvoid caused by γ-irradiation. The stress concentration was generated in fibers owing to the existence of microvoid when they were stretched. And it was easier to generate fracture for fibers with larger microvoid.^23^ Thus the decrease of microvoid size by γ-rays also caused the improvement of mechanical property for CFs.

## 4. Conclusions

In summary, average microvoid radius of CFs decreased gradually from 4.8406 nm for pristine fibers to 3.6868 nm (ECP) and 3.4223 nm (Ar), indicating that microvoid size of CFs was decreased by γ-irradiation owing to annealing and rearrangement. The γ-rays also decreased the *d*_002_ but enhanced mechanical property of CFs. According to evolution trend of microvoid, *d*_002_ and mechanical property for CFs in different active media, we concluded that microvoid decrease by γ-irradiation was also the one of primary cause for improvement of mechanical property. The microvoid evolution of γ-irradiated CFs was investigated by SAXS, and pertinence of microvoid and mechanical property in γ-irradiation had also been evaluated, which enriched mechanism of mechanical property evolution for γ-irradiation CFs and was conducive to improving the mechanical property of γ-irradiated CFs continuously.

## Conflicts of interest

There are no conflicts to declare.

## Supplementary Material
